# Sites of skin inflammation recurrence in patients with SLE: an analysis of clinical trial data

**DOI:** 10.1136/lupus-2025-001916

**Published:** 2026-02-15

**Authors:** Elizabeth Peterknecht, Marco Branco, John A Reynolds

**Affiliations:** 1 Department of Inflammation and Ageing, School of Infection, Inflammation and Immunology, College of Medicine and Health, University of Birmingham, Birmingham, UK; 2 Rheumatology Department, Sandwell and West Birmingham NHS Trust, Birmingham, UK

**Keywords:** Clinical Trial, Inflammation, Outcome Assessment, Health Care

 Skin manifestations are common in patients with SLE. A recent study reported increased site-specific recurrence of cutaneous lupus erythematosus (CLE).^1^ While this study included a proportion of patients with SLE, we wanted to confirm whether site-specific recurrence occurred in a large group of patients with SLE.

We analysed patients in the standard of care (SoC) arms from three randomised controlled trials (RCTs) of anifrolumab in patients with active SLE (NCT02446899, NCT02446912 and NCT01438489). All participants met the 1982 revised American College of Rheumatology classification criteria for SLE. Skin disease was captured using the Cutaneous Lupus Erythematosus Disease Area and Severity Index (CLASI).^2^ We used the erythema subsection of CLASI to define active disease; in each of the 13 body areas, a score of ≥1 was considered active disease. Skin remission was defined as an erythema score of 0 in all 13 domains and recurrence as a CLASI erythema score of ≥1 in any of the 13 domains following at least one remission visit.

We analysed 437 patients of whom 405 (92.7%) were female with median (IQR) disease duration of 5.41 (2.12–9.92) years. Most patients (263, 60.2%) were White and active skin disease was present in (371, 84.9%) at baseline (see online supplemental table 1). The most common site of inflammation at baseline was malar rash in 257 (58.8%) patients, followed by the rest of face (184, 42.1%) and the V area (158, 36.2%). Relatively few patients had active inflammation on the abdomen (23, 5.2%) or feet (30, 6.9%) (see online supplemental table 2).

In those patients with skin involvement at baseline, 182/371 (49.1%) entered skin remission. Of these patients, 54/182 (29.7%) had recurrence. In unadjusted logistic regression models, the site of skin involvement at first recurrence was significantly associated with involvement of the same site at baseline in 9 of the 13 sites. In models adjusted for age band, sex and ethnicity, the association between site of recurrence and site involved at baseline remained in the nine domains (see figure 1 and online supplemental table 3).

**Figure 1:**
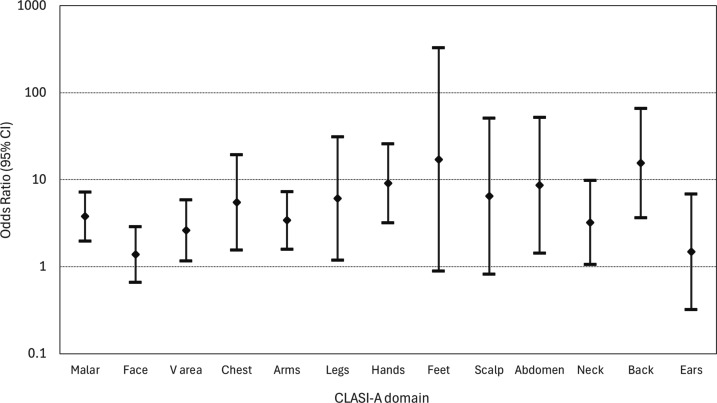
The figure shows the OR and 95% CI for involvement of each of the 13 skin sites if that site was involved at baseline, adjusted for age band, sex and ethnic group. The diamond shows the OR and the whiskers show the 95% CI. CLASI, Cutaneous Lupus Erythematosus Disease Area and Severity Index.

This study has identified that skin inflammation in patients with SLE is more likely to occur as sites of previous inflammation. In this study, all patients had SLE confirmed by meeting classification criteria and were treated with SoC. While ultraviolet (UV) light exposure is an important trigger of SLE skin activity, this is unlikely to fully explain our observations as UV exposure is not typically equal across all body areas. Strengths of this study are that the three RCTs included patients from a broad range of sites globally and a range of ethnic backgrounds.

Our study has some limitations. The number of patients with erythema recurrence was relatively small and so we lack statistical power to identify associations in skin domains less commonly affected. We also used a stringent definition of remission which limited the number of patients available to analyse recurrence but ensured that all recurrences occurred after complete remission. To capture any new inflammation at any site, our definition (of an erythema score of 1) was more generous than the definition of clinical flare^3^; there were insufficient numbers of patients to study a clinical definition of flare. The nature of the data meant that we had to consider recurrence in body areas and were unable to determine whether the recurrence was within the same lesion. However, others have confirmed recurrence within lesions by analysis of photographic documentation.^1^ While we were unable to confirm the subtype of CLE in our data, it has been reported that most discoid lupus erythematosus recurrence affects the same area, while subacute cutaneous lupus erythematosus affects both old and new areas.^1^ Our analysis was also agnostic to the SoC treatment that patients received.

Recurrence of CLE lesions at affected sites in patients with SLE is common. Environmental triggers such as UV exposure may be important, but recurrence across all body sites suggests tissue priming at the cellular or molecular level may occur following inflammation resolution. While tissue-resident memory T (Trm) cells are increased in CLE lesions, their cognate antigens are unknown.^4^ Modification of Trm responses may be an important approach to reducing skin flare in patients with SLE.

## Supplementary material

10.1136/lupus-2025-001916 online supplemental file 1
