# Supplementary material for: Sites of skin inflammation recurrence in patients with SLE: an analysis of clinical trial data
Source: Lupus Sci Med. 2026 Feb 15;13(1):e001916. doi: 10.1136/lupus-2025-001916 (PMC12911678; doi:10.1136/lupus-2025-001916)
Supplement: online supplemental file 1 [file lupus-13-1-s001.docx]

Supplementary Table S1: Characteristics of the patients at study screening

|  | N(%) or median (IQR) |
| --- | --- |
| Age band  ≤ 20  20 ≤ 40  40 ≤ 60  60+  Missing | 11 (2.52%)  202 (46.22%)  194 (44.39%)  27 (6.18%)  3 (0.69%) |
| Female | 405 (92.68%) |
| Race  White  Black or African American  Asian  Other  Mixed  American Indian or Alaska Native  Missing | 263 (60.18%)  60 (13.73%)  40 (9.15%)  62 (14.19%)  1 (0.23%)  2 (0.46%)  9 (2.06%) |
| Disease duration (years) | 5.41 (2.12, 9.92) |
| Baseline CLASI score | 6 (3, 10) |
| Patients with CLASI=0 at baseline | 17 (4.99%) |
| Baseline global BILAG score | 18 (16, 21) |
| Baseline SLEDAI score | 10 (8, 13) |

Supplementary Table S2: Sites of active inflammation at baseline

| Site | Baseline involvement (n%)  (n=437) |
| --- | --- |
| Malar | 257 (58.8%) |
| Rest of face | 184 (42.1%) |
| V area | 158 (36.2%) |
| Chest | 97 (22.2%) |
| Arms | 138 (31.6%) |
| Legs | 49 (11.2%) |
| Hands | 83 (19.0%) |
| Feet | 30 (6.8%) |
| Scalp | 84 (19.2%) |
| Abdomen | 23 (5.3%) |
| Neck | 94 (21.5%) |
| Back | 46 (10.5%) |
| Ears | 63 (14.4%) |

Supplementary Table S3: Logistic regression models of site of skin flare according to whether or not that site was involved at baseline

|  | Unadjusted |  | Adjusted 1* |  |
| --- | --- | --- | --- | --- |
|  | OR | 95% CI | OR | 95% CI |
| Malar | **3.561** | **1.934, 6.553** | **3.783** | **1.983, 7.215** |
| Face | 1.711 | 0.860, 3.404 | 1.387 | 0.666, 2.888 |
| V area | **2.750** | **0.1283, 5.849** | **2.624** | **1.164, 5.911** |
| Chest | **8.039** | **2.826, 22.862** | **5.483** | **1.553, 19.353** |
| Arms | **3.604** | **1.799, 7.224** | **3.409** | **1.596, 7.282** |
| Legs | **4.636** | **1.150, 18.684** | **6.113** | **1.198, 31.196** |
| Hands | **7.536** | **2.967, 19.143** | **9.118** | **3.205, 25.941** |
| Feet | 8.428 | 0.831, 85.480 | 17.160 | 0.896, 328.608 |
| Scalp | 3.962 | 0.639, 24.567 | 6.513 | 0.825, 51.389 |
| Abdomen | **12.611** | **3.196, 49.763** | **8.661** | **1.433, 52.340** |
| Neck | **4.972** | **1.8777, 13.167** | **3.230** | **1.0638, 9.8068** |
| Back | **20.272** | **6.045, 67.982** | **15.563** | **3.657, 66.227** |
| Ears | 3.888 | 0.984, 15.367 | 1.487 | 0.321, 6.898 |

*adjusted for age band, sex and ethnic group
